# Risk factors for squamous cell carcinoma of the oesophagus in women: a case–control study

**DOI:** 10.1054/bjoc.2001.2147

**Published:** 2001-11

**Authors:** L Sharp, C E D Chilvers, K K Cheng, P A McKinney, R F A Logan, P Cook-Mozaffari, A Ahmed, N E Day

**Affiliations:** Department of Medicine & Therapeutics, University of Aberdeenw; Division of Public Health Medicine and Epidemiology, University of Nottingham; Department of Public Health and Epidemiology, University of Birmingham; Information and Statistics Division, NHS in Scotland; Division of Public Health and Primary Health Care, University of Oxford; Department of Community Medicine, University of Cambridge

**Keywords:** oesophagus, cancer, case–control, aetiology

## Abstract

Oesophageal cancer rates in women in the UK are more than 3 times higher than in most other European populations. A population-based matched case–control study of histologically confirmed squamous cell carcinoma of the oesophagus in women was carried out in 4 regions in England and Scotland. Interviews were carried out in hospital or at home and topics included: smoking; alcohol; tea and coffee consumption; medical and obstetric history; and diet. Response rates were 62% for cases and 65% for first-chosen controls. There were 159 case–control pairs. Significant results were found for: eating salads (odds ratio (OR) 0.42, 95% CI 0.20–0.92 in the highest quartile of consumption) and a light (as distinct from no) breakfast (OR 0.18, 95% CI 0.07 – 0.48) were protective; quantity of tea was a risk factor and there was a significant positive trend with temperature at which hot drinks were consumed (*P* = 0.03). Alcohol consumption was unrelated to risk, but there was a significant trend with years of smoking (*P* = 0.015). A protective effect of aspirin consumption was confined to the English centres (OR 0.08, 95% CI 0.01–0.56). Comparison with a parallel study of adenocarcinoma indicated a common protective effect of a healthy diet but otherwise distinct risk factors. © 2001 Cancer Research Campaign http://www.bjcancer.com


*British Journal of Cancer* (2001) **85**(11), 1667–1670
© 2001 Cancer Research Campaign
doi: 10.1054/ bjoc.2001.2147, available online at http://www.idealibrary.com on IDEAL

http://www.bjcancer.com

### Risk factors for squamous cell carcinoma of the oesophagus in women: a case–control study

**L Sharp[1], CED Chilvers[2], KK Cheng[3], PA McKinney[4], RFA Logan[2], P Cook-Mozaffari[5], A Ahmed[6] and NE Day[6]**

[1]Department of Medicine & Therapeutics, University of Aberdeen; [2]Division of Public Health Medicine and Epidemiology, University of Nottingham; [3]Department of Public Health and Epidemiology, University of Birmingham; [4]Information and Statistics Division, NHS in Scotland; [5]Division of Public Health and Primary Health Care, University of Oxford; [6]Department of Community Medicine, University of Cambridge

**Summary** Oesophageal cancer rates in women in the UK are more than 3 times higher than in most other European populations. A population-based matched case–control study of histologically confirmed squamous cell carcinoma of the oesophagus in women was carried out in 4 regions in England and Scotland. Interviews were carried out in hospital or at home and topics included: smoking; alcohol; tea and coffee consumption; medical and obstetric history; and diet. Response rates were 62% for cases and 65% for first-chosen controls. There were 159 case–control pairs. Significant results were found for: eating salads (odds ratio (OR) 0.42, 95% CI 0.20–0.92 in the highest quartile of consumption) and a light (as distinct from no) breakfast (OR 0.18, 95% CI 0.07 – 0.48) were protective; quantity of tea was a risk factor and there was a significant positive trend with temperature at which hot drinks were consumed ($P = 0.03$). Alcohol consumption was unrelated to risk, but there was a significant trend with years of smoking ($P = 0.015$). A protective effect of aspirin consumption was confined to the English centres (OR 0.08, 95% CI 0.01–0.56). Comparison with a parallel study of adenocarcinoma indicated a common protective effect of a healthy diet but otherwise distinct risk factors. © 2001 Cancer Research Campaign http://www.bjcancer.com

**Keywords**: oesophagus; cancer; case–control; aetiology

Oesophageal cancer rates in women in the UK are more than 3 times higher than in most other European populations (Macfarlane and Boyle, 1994). Between 1956–60 and 1986–90, mortality from oesophageal cancer in women in England and Wales and in Scotland increased by more than 30% (Cheng and Day, 1992). This increase has mainly been due to an increase in adenocarcinomas, but there has also been some increase in squamous cell cancers both in Britain (Powell and Allum, 1992; Dodaran et al, 2001), and in Scotland where increases in incidence have recently been observed in both subtypes for men and women (McKinney et al, 1995; Brewster et al, 2000). Squamous cell tumours are still the most common subtype in the UK. Both squamous cell carcinoma and adenocarcinoma of the oesophagus have an extremely poor prognosis and, as there seems little prospect of improvement in early detection or treatment, a better understanding of their aetiology may suggest opportunities for primary prevention. We have already reported the results for adenocarcinoma of the oesophagus (Cheng et al, 2000), which suggested that high body mass index (BMI) and low consumption of fruit were associated with an increased risk, and that there was a protective effect of breast-feeding. In this paper we report the results for squamous cell carcinoma of the oesophagus.

For squamous cell carcinoma, the 2 major risk factors identified in the male population are alcohol and tobacco consumption (Macfarlane and Boyle, 1994) but these are unlikely to explain the high incidence of the disease in women. Studies elsewhere in the world have suggested that chronic injury to the oesophagus from consumption of hot beverages (Victoria et al, 1989; de Stefani et al, 1990), and dietary deficiencies may play a part (Cheng and Day, 1996). These may also help to explain the high incidence in women in the UK.

*Received 12 March 2001*
*Revised 5 September 2001*
*Accepted 10 September 2001*

*Correspondence to:* L Sharp

### MATERIALS AND METHODS

These are described in full elsewhere (Cheng et al, 2000). Briefly, a population based case–control study was carried out in 3 regions in England and in eastern Scotland, following approval from the relevant local research ethics committees.

Cases were women aged less than 75 years at diagnosis (under 80 in Trent), diagnosed between 1993 and 1996, with histologically confirmed squamous cell carcinomas of the oesophagus. One control was matched to each case by age (within 5 years) and by general practice. Interviews were carried out in hospital or at home, and topics included smoking, alcohol, tea and coffee consumption, medical and obstetric history, and diet.

Conditional logistic regression was carried out using STATA (StataCorp, 1999), with dose–response tested for trend (Breslow and Day, 1980). All variables were examined in a univariate analysis. Those that had a significance level of at least 0.1 were adjusted for in the multivariate analysis. Interactions with ‘Centre’ (England or Scotland) were also examined because of the known dietary differences between the 2 countries. Where there was a significant interaction this was also adjusted for in the multivariate analysis.

### RESULTS

Of 416 eligible patients with any histological type of oesophageal cancer, 256 (62%) were interviewed. The response rate for first-choice controls was 65%. There were 159 cases of squamous cell carcinoma, 86 in the English centres and 73 in Scotland.

**Table 1** Characteristics of respondents by case–control status

| | No[a] (%) of | | Unadjusted OR | 95% CI | *P* (trend) |
|---|---|---|---|---|---|
| | cases | controls | | | |
| Age | | | | | |
| < 50 | 9 ( 5.7) | 8 ( 5.0) | | | |
| 50–59 | 23 (14.5) | 22 (13.8) | | | |
| 60–69 | 58 (36.5) | 63 (39.6) | | | |
| ≥ 70 | 69 (43.4) | 66 (41.5) | | | |
| Social class | | | | | |
| I + II | 46 (28.9) | 47 (29.6) | 1 | | |
| IIIA | 9 ( 5.7) | 18 (11.3) | 0.52 | 0.22–1.24 | |
| IIIB | 64 (40.3) | 62 (39.0) | 1.19 | 0.65–2.15 | |
| IV + V | 37 (23.3) | 29 (18.2) | 1.51 | 0.75–3.06 | |
| armed forces | 3 ( 1.9) | 3 ( 1.9) | 1.03 | 0.20–5.38 | 0.493[b] |
| Marital status | | | | | |
| married or living as married | 87 (55.1) | 97 (61.4) | 1 | | |
| other | 71 (44.9) | 61 (38.6) | 1.37 | 0.83–2.25 | 0.211 |
| Further training | | | | | |
| none | 129 (81.1) | 118 (74.2) | 1 | | |
| nursing/teaching/secretarial/other | 28 (17.6) | 35 (22.0) | 0.66 | 0.35–1.23 | |
| university | 2 ( 1.3) | 6 ( 3.8) | 0.28 | 0.06–1.44 | 0.059 |

[a]Excludes pairs where one or more subjects have missing data. [b]Trend across first 4 categories – I + II, IIIA, IIIB, IV + V.

Cases were slightly more likely to be of lower social class and to have had no further training after leaving school than controls (Table 1) but neither trend was statistically significant. Cases were slightly less likely than controls to be married or living as married.

The dietary factors (Table 2) associated with a significantly decreased odds ratio (OR) were eating a ‘light’ breakfast (defined as other than the traditional British cooked breakfast) (adjusted OR 0.18, 95% CI 0.07–0.48), and eating salads (trend with increasing salad consumption $P = 0.005$; adjusted OR 0.42, 95% CI 0.20–0.92 in the highest quartile of consumption). A slimming diet, and meal patterns (classed as nutritionally good, moderate, or bad), total fruit and fruit juice consumption all showed an association with squamous cell carcinoma of the oesophagus in the unadjusted analysis which disappeared after adjustment. Quantity of tea consumed was of borderline significance in the adjusted analysis (highest tertile vs never/<1 decilitre per day OR = 3.36, 95% CI 0.99–11.29, *P* for trend = 0.052). There was a significant increasing trend in risk with increasing temperature at which tea or coffee is drunk ($P = 0.03$).

Alcohol consumption was unrelated to squamous cell cancer. There were positive trends with total years of smoking and pack years of smoking (Table 2).

Reproductive variables (number of children, breast feeding) were unrelated to risk of squamous cell carcinoma (data not shown). Of the medical and drug history items (which included anaemia, diabetes, indigestion, obesity, ingestion of chemicals and laxative use), none showed any effect on risk apart from aspirin which showed a protective effect. Based on 10 (6.3%) cases and 19 (12.0%) controls reporting daily use of aspirin for at least a month, there was a significant interaction between aspirin and centre. The protective effect was significant only for the English centres (OR 0.08, 95% CI 0.01–0.56 for the English centres and OR 0.77, 95% CI 0.21–2.94 for Scotland).

### DISCUSSION

An important finding in this study of squamous cell carcinomas of the oesophagus in women is that alcohol consumption is not a major risk factor. Levels of alcohol consumption in this study were relatively low with only 5% of control women and 7% of cases reporting consumption greater than the recommended weekly limit for women of 14 units. In studies largely conducted among men in Western Europe and North America, it has been estimated that at least 90% of the risk of oesophageal cancer can be attributed to alcohol and tobacco consumption, and risks associated with alcohol consumption are greater than those associated with smoking (see Muñoz and Day, 1996 and references therein). The possibility cannot be excluded that the participants in our study under-reported their alcohol intake. We attempted to minimise potential for differential and non-differential under-reporting. Participants were not made aware that one of the study hypotheses pertained to alcohol. Our trained study interviewers used a structured interview schedule to obtain information on alcohol intake from cases and controls. Therefore it seems unlikely that there would have been under-reporting of sufficient magnitude to entirely account for our null finding.

Smoking and alcohol are frequently correlated but in the absence of any effect for alcohol, risks were clearly increased for women who smoked. A dose–response effect was evident, with a 2- to 3-fold increase in risk for the highest levels of consumption. In England a decrease in cigarette smoking for men is not paralleled by a decrease in women (Department of Health, 1998) and therefore the risk of oesophageal cancer in this population is likely to remain high. Recent data from Scotland show that the proportion of women who smoked in 1998 (33%) was lower than in 1995 (36%) (Boreham, 2000) indicating that risk is likely to diminish in this population. However a strong association between lower social class and high levels of smoking may mean that for those in the more deprived groups with poor levels of nutrition the risk of oesophageal cancer may still be raised.

There is substantial evidence for an association between nutritional factors and oesophageal cancer (Cheng and Day, 1996). Our finding of a protective effect of a light breakfast as well as for fruit consumption may be a marker of a ‘healthy’ diet. However, our results also suggest that having some kind of breakfast is better

 *© 2001 Cancer Research Campaign*

**Table 2** Components of diet, and history of cigarette consumption associated with squamous cell carcinoma of oesophagus

| Variable | No[b] (%) of cases | No[b] (%) of controls | Unadjusted OR | Unadjusted 95% CI | Unadjusted P (trend) | Adjusted[a] OR | Adjusted[a] 95% CI | Adjusted[a] P (global) | Adjusted[a] P (trend) |
|---|---|---|---|---|---|---|---|---|---|
| Slimming diet | | | | | | | | | |
| no | 156 (98.1) | 144 (90.6) | 1 | – | | 1 | – | | |
| yes | 3 ( 1.9) | 15 ( 9.4) | 0.20 | 0.06–0.69 | 0.005 | 0.29 | 0.07–1.27 | 0.078 | – |
| Breakfast | | | | | | | | | |
| no breakfast | 31 (19.5) | 17 (10.7) | 1 | – | | 1 | – | | |
| cooked breakfast | 47 (29.6) | 34 (21.4) | 0.75 | 0.35–1.60 | | 0.40 | 0.15–1.08 | | |
| other type of breakfast | 81 (50.9) | 108 (67.9) | 0.39 | 0.20–0.78 | 0.002 | 0.18 | 0.07–0.48 | 0.0004 | 0.0001 |
| Meal pattern: midday and evening | | | | | | | | | |
| good | 23 (14.5) | 37 (23.3) | 1 | – | | 1 | – | | |
| moderate | 109 (68.6) | 99 (62.3) | 1.85 | 1.00–3.41 | | 1.18 | 0.56–2.46 | | |
| bad | 27 (17.0) | 23 (14.5) | 2.10 | 0.92–4.81 | 0.066 | 0.86 | 0.31–2.40 | 0.698 | 0.825 |
| All salad (times per week) | | | | | | | | | |
| 0–6.44 | 58 (36.7) | 39 (24.7) | 1 | – | | 1 | – | | |
| 6.45–11.46 | 48 (30.4) | 39 (24.7) | 0.83 | 0.51–1.54 | | 0.87 | 0.46–1.67 | | |
| 11.47–17.11 | 22 (13.9) | 39 (24.7) | 0.51 | 0.19–0.74 | | 0.28 | 0.12–0.68 | | |
| ≥ 17.12 | 30 (19.0) | 41 (26.0) | 0.45 | 0.20–0.94 | 0.005 | 0.42 | 0.20–0.92 | 0.009 | 0.005 |
| Total fruit (times per week) | | | | | | | | | |
| 0–12.00 | 58 (36.7) | 39 (24.7) | 1 | – | | 1 | – | | |
| 12.01–18.04 | 42 (26.6) | 41 (26.0) | 0.63 | 0.41–1.31 | | 0.72 | 0.35–1.49 | | |
| 18.05–25.72 | 32 (20.3) | 40 (25.3) | 0.55 | 0.33–1.07 | | 0.81 | 0.37–1.80 | | |
| ≥ 25.73 | 26 (16.5) | 38 (24.0) | 0.38 | 0.24–0.91 | 0.012 | 0.64 | 0.25–1.67 | 0.764 | 0.394 |
| Fruit juice | | | | | | | | | |
| never | 94 (59.5) | 85 (53.8) | 1 | – | | 1 | – | | |
| < 1/day | 49 (31.0) | 44 (27.9) | 0.93 | 0.56–1.92 | | 0.86 | 0.45–1.66 | | |
| 1/day | 5 ( 3.2) | 10 ( 6.3) | 0.71 | 0.29–1.50 | | 0.72 | 0.18–2.84 | | |
| > 1/day | 10 ( 6.3) | 19 (12.0) | 0.41 | 0.23–0.96 | 0.016 | 0.84 | 0.29–2.42 | 0.946 | 0.628 |
| Tea (volume per day) | | | | | | | | | |
| never/< 1/day | 10 ( 6.3) | 17 (10.8) | 1 | – | | 1 | – | | |
| ≤ 6 dcl | 42 (26.6) | 48 (30.4) | 1.21 | 0.57–3.85 | | 2.33 | 0.62– 8.86 | | |
| 7–11 dcl | 47 (29.8) | 48 (30.4) | 1.31 | 0.65–4.13 | | 2.99 | 0.85–10.56 | | |
| ≥ 12 dcl | 59 (37.3) | 45 (28.5) | 1.49 | 0.90–5.42 | 0.053 | 3.36 | 0.99–11.29 | 0.198 | 0.052 |
| Temperature of tea or coffee | | | | | | | | | |
| very/burning hot | 50 (32.1) | 53 (34.0) | 1 | – | | 1 | – | | |
| hot | 81 (51.9) | 72 (46.2) | 1.21 | 0.71–2.01 | | 0.75 | 0.38–1.47 | | |
| warm | 25 (16.0) | 31 (19.9) | 1.04 | 0.41–1.71 | 0.790 | 0.34 | 0.13–0.88 | 0.066 | 0.030 |
| Smoking status | | | | | | | | | |
| never smoked | 55 (34.8) | 58 (36.7) | 1 | – | | 1 | – | | |
| ex-smoker | 41 (26.0) | 60 (38.0) | 0.65 | 0.37–1.15 | | 0.48 | 0.25–0.93 | | |
| current smoker | 62 (39.2) | 40 (25.3) | 1.91 | 1.06–3.44 | 0.081 | 1.34 | 0.66–2.70 | 0.019 | 0.779 |
| Total years of smoking | | | | | | | | | |
| never smoked | 55 (34.8) | 58 (36.7) | 1 | – | | 1 | – | | |
| ex-smoker | 41 (26.0) | 60 (38.0) | 0.63 | 0.36–1.12 | | 0.46 | 0.24–0.90 | | |
| ≤ 37.68 | 10 ( 6.3) | 12 ( 7.6) | 0.82 | 0.26–2.56 | | 0.39 | 0.10–1.53 | | |
| 37.69–48.57 | 21 (13.3) | 13 ( 8.2) | 1.94 | 0.80–4.72 | | 0.92 | 0.29–2.97 | | |
| ≥ 48.58 | 31 (19.6) | 15 ( 9.5) | 2.48 | 1.11–5.51 | 0.001[10] | 2.35 | 0.91–6.03 | 0.009 | 0.015[c] |
| Pack-years | | | | | | | | | |
| never smoked | 55 (34.8) | 58 (36.7) | 1 | – | | 1 | – | | |
| ex-smoker | 41 (26.0) | 60 (38.0) | 0.65 | 0.37–1.14 | | 0.48 | 0.25–0.93 | | |
| ≤ 16.63 | 10 ( 6.3) | 11 ( 7.0) | 1.02 | 0.37–2.86 | | 1.05 | 0.32–3.43 | | |
| 16.64–32.02 | 22 (13.9) | 15 ( 9.5) | 2.04 | 0.86–4.88 | | 1.60 | 0.56–4.60 | | |
| ≥ 32.03 | 30 (19.0) | 14 ( 8.9) | 2.36 | 1.10–5.07 | 0.002[11] | 1.35 | 0.53–3.43 | 0.083 | 0.079[d] |
| Average weekly alcohol consumption over lifetime (units/week) | | | | | | | | | |
| non-drinker | 57 (36.5) | 49 (31.4) | 1 | – | | 1 | – | | |
| < 2 | 47 (30.1) | 52 (33.3) | 0.80 | 0.47–1.37 | | 0.81 | 0.42–1.56 | | |
| 2–13.99 | 41 (26.3) | 47 (30.1) | 0.75 | 0.42–1.33 | | 0.72 | 0.34–1.53 | | |
| ≥ 14 | 11 ( 7.1) | 8 ( 5.1) | 1.23 | 0.44–3.37 | 0.629 | 0.86 | 0.25–2.95 | 0.840 | 0.454 |

[a]Adjusted for slimming diet, breakfast, salad, years smoking, regular use of aspirin, aspirin*centre, and temperature of tea/coffee. [b]Excludes pairs where one or more subjects have missing data. [c]Trend test based on 4 categories: never plus ex-smoker, ≤ 37.68, 37.69–48.57, ≥ 48.58 years. [d]Trend test based on 4 categories: never plus ex-smoker, ≤ 16.63, 16.64–32.02, ≥ 32.03 pack years.

*© 2001 Cancer Research Campaign*

than having none at all, so another explanation might be that eating breakfast has some physiological effect which results in a reduced risk of developing this tumour. One such possibility could be the effect of food on morning gastric reflux. These potential explanations for our observation with regard to breakfast are speculative; this issue requires further investigations.

Although the increasing trend in risk with quantity of tea consumed is of borderline significance only, the risks are substantial (over 3-fold at the highest level of consumption), and there is an almost 3-fold greater risk in those who drink beverages ‘very or burning’ hot compared to warm. Given the possibility of a large measurement error, the real risks could be substantially larger and taken with the effect of smoking, this seems the most likely explanation of the relatively high rates of squamous cell cancer of the oesophagus in women in the UK compared to other European populations.

In the early 1990s a large prospective study from the US identified a protective effect for gastrointestinal cancers including those of the oesophagus associated with the long-term use of aspirin (Thun et al, 1993). More recently Langman et al (2000) using data from a UK primary care database, and Farrow et al (1998) in a case–control study in the USA have reported protective effects of nonsteroidal anti-inflammatory drugs. Farrow et al found the effect for both squamous cell and adenocarcinoma of the oesophagus. Overall the findings from our study are consistent with this but the negative association is only significant for the English centres. The reasons for this remain unclear. We have previously reported a non-significant protective effect with respect to adenocarcinoma (OR = 0.67, 95% CI 0.27–1.63) (Cheng et al, 2000). The question we asked was a simple one relating to ever use of aspirin regularly for a month. This differs from the definition used by Langman et al which relates to at least 7 prescriptions for anti-inflammatory drugs in the 1–3 years prior to diagnosis.

The parallel studies of adenocarcinoma (Cheng et al 2000) and squamous cell carcinoma in the same centres by the same study team provides an opportunity to compare and contrast the findings for each tumour type. In the final model for squamous cell carcinoma the following variables were statistically significant: type of breakfast; salad consumption; temperature of tea or coffee; total years of smoking and aspirin use, which had a significant interaction with Centre. The final model for adenocarcinoma showed risk increased significantly with high body mass index at 20 years of age ($P$ for trend = 0.002) and that higher intake of fruit (times per week, $P$ for trend = 0.002) and breastfeeding ($P$ for trend = 0.005) were associated with reduced risk. This suggests differences in aetiology of the 2 tumour types. In common is a protective effect of a ‘healthy diet’ though the significant variables were not identical. For both a healthy diet might be a good preventive strategy, with weight control also being important in adenocarcinoma. In squamous cell carcinoma smoking cessation and care not to drink beverages at too high a temperature are additional strategies. Additionally, if the association with aspirin is a true one, it offers the possibility of an intervention strategy in target populations, such as smokers.

### ACKNOWLEDGEMENTS

We thank the study co-ordinators and interviewers: Sandra Bonney, Gwyn Campion, Jane Davies, Gail Faulkner, Marie Fleming, Jean Fraser, Margaret Rayner, Doreen Refaat, Cherry Robertson, Jane Shearer, and Rosemary Wylie. We also thank the pathologists, clinicians and general practitioners who assisted us in the study. The study was funded by the Chief Scientist Office, Scottish Office; the LORS, East Anglia; Special Trustees of the Nottingham University Hospitals, Trent; and the Medical Research Council, Oxford.

### REFERENCES

Boreham R (2000) *The Scottish Health Survey 1998: Volume 1: Chapter 8 Smoking*. Scottish Executive Health Department

Breslow NE and Day NE (1980) *Statistical methods in cancer research: vol. 1. The analysis of case–control studies*. IARC Scientific publications, No 32. IARC: Lyon

Brewster DH, Fraser LA, McKinney PA and Black RJ (2000) Socioeconomic status and risk of adenocarcinoma of the oesophagus and cancer of the gastric cardia in Scotland. *Brit J Cancer* **83**: 387–390

Cheng KK and Day NE (1992) Oesophageal cancer in Britain. *Brit Med J* **304**: 711

Cheng KK and Day NE (1996) Nutrition and oesophageal cancer. *Cancer Causes Control* **7**: 33–40

Cheng KK, Sharp L McKinney PA, Logan RFA, Chilvers CED, Cook-Mozaffari P, Ahmed A and Day NE (2000) The preventability of oesophageal adenocarcinoma in women: a case control study. *Brit J Cancer* **83**: 127–132

Department of Health (1998) *Statistics on smoking: England 1976 to 1996*. Statistical Bulletin 1998/25. London: Department of Health de Stefani, Muñoz N, Estēve J, Vasallo A, Victoria CG and Teuchmann S (1990) Mate drinking, alcohol, tobacco, diet and esophageal cancer in Uruguay. *Cancer Res* **50**: 426–431

Dodaran MS, Silcocks PB and Logan RFA (2001) Continuing rise in incidence of oesophageal adenocarcinoma in England and Wales 1971–1998. *Gut* **48**, Supplement 1 (in press)

Farrow DC, Vaughan TL, Hansten PD, Stanford JL, Risch HA et al (1998) Use of aspirin and other nonsteroidal anti-inflammatory drugs and risk of esophageal and gastric cancer. *Cancer Epidemiol Biomark Prevent* **7**: 97–102

Langman MJS, Cheng KK, Gilman EA and Lancashire RJ (2000) Effect of anti-inflammatory drugs on overall risk of common cancer: case-control study in general practice research database. *Brit Med J* **320**: 1642–1646

Macfarlane GJ and Boyle P (1994) The epidemiology of oesophageal cancer in the UK and other European countries. *J Royal Soc Med* **87**: 334–337

Muñoz N and Day NE (1996) Esophageal Cancer. In Schottenfeld D and Fraumeni JF Jr (Eds) Cancer Epidemiology and Prevention. Oxford University Press, New York Oxford

Powell J and Allum WH (1992) Oesophageal cancer in Britain. *Brit Med J* **304**: 1380

StataCorp (1999) Stata Statistical Software: Release 6.0. College Station, Texas: Stata Corporation

Victoria CG, Muñoz N, Day NE, Barcelos LB, Peccin DA and Braga NM (1987) Hot beverages and oesophageal cancer in Southern Brazil: a case-control study. *Int J Cancer* **39**: 710–716

*© 2001 Cancer Research Campaign*
